# Moderate intensity intermittent lifestyle physical activity is associated with better executive function in older adults

**DOI:** 10.3389/fspor.2024.1393214

**Published:** 2024-05-21

**Authors:** Emily MacDonald, Elisabeth G. Morrison, Madeline E. Shivgulam, Liam P. Pellerine, Derek S. Kimmerly, Nick W. Bray, Said Mekari, Myles W. O’Brien

**Affiliations:** ^1^ Department of Neuroscience, Faculty of Science, Dalhousie University, Halifax, NS, Canada; ^2^ School of Kinesiology, Acadia University, Wolfville, NS, Canada; ^3^ Division of Kinesiology, School of Health and Human Performance, Faculty of Health, Dalhousie University, Halifax, NS, Canada; ^4^ Faculty of Medicine, Memorial University of Newfoundland, St. John’s, NL, Canada; ^5^ Department of Medicine, Université de Sherbrooke, Sherbrooke, QC, Canada; ^6^ Centre de Formation Médicale Du Nouveau-Brunswick, Université de Sherbrooke, Moncton, NB, Canada

**Keywords:** physical activity intensity, short bout physical activity, cognitive aging, cognitive decline, Trail-Making-Task

## Abstract

Executive functions are among the first cognitive abilities to decline with age and age-related executive function slowing predisposes older adults to cognitive disorders and disease. Intermittent Lifestyle Physical Activity (ILPA) reflects brief, unplanned activity that occurs during routine daily activities and is operationalized as activity bouts <60s. Our understanding of short bouts of habitual physical activity and executive functions is limited. We tested the hypothesis that greater amounts of ILPA in moderate and vigorous intensity domains would be associated with better executive function in older adults. Forty older adults (26 females, 68 ± 6, >55 years; body mass index: 26.6 ± 4.3 kg/m^2^) completed a Trail-Making-Task and wore an activPAL 24-hr/day for 6.2 ± 1.8-days. For each intensity, total time and time spent in bouts <60 s were determined. Trail A (processing speed) and Trail B (cognitive flexibility) were completed in 25.8 ± 8.2 s and 63.2 ± 26.2 s, respectively. Non-parametric Spearman's rank correlations report that moderate ILPA (3.2 ± 3.2 min/day) and total-moderate physical activity (20.1 ± 16.0 min/day) were associated with faster Trail A (total-moderate physical activity: *ρ*=−0.48; moderate-ILPA: *ρ* = −0.50; both, *p *< 0.003) and Trail B time (total-moderate physical activity: *ρ* = 0.36; moderate-ILPA: *ρ *= −0.46; both, *p *< 0.020). However, the results show no evidence of an association with either vigorous physical activity or light physical activity (total time or ILPA bouts: all, *p *> 0.180). Moderate physical activity accumulated in longer bouts (>60 s) was not associated with Trail B time (*p = *0.201). Therefore, more total moderate physical activity and shorter bouts (<60 s) may result in better executive functions in older adults.

## Introduction

It is well-established that cognitive decline is a critical health risk to the aging population, with ∼50% of adults ≥85 years old exhibiting evidence of cognitive impairments (1). Cognitive decline is initially characterized by the deterioration of executive functions, which are a set of cognitive processes encompassing skills such as working memory, cognitive flexibility, planning, and regulating behavior (2). Worsening executive function is recognized as an early predictor for many neurological conditions, including dementia and Alzheimer's disease (3, 4). There is increasing support for regular physical activity as a useful strategy for slowing this cognitive decline, specifically in older adults (5). Our understanding of the impact between the intensity and duration of physical activity with executive functions in older adults is unclear but could prove useful in helping guide strategies that prevent the development of cognitive damages associated with age.

The World Health Organization physical activity guidelines recommend that adults >65 years old accumulate at least 150 min of moderate-to-vigorous physical activity per week (6). Habitual physical activity refers to daily leisure activities conducted at home or work (7). While laboratory-based studies have demonstrated that engaging in aerobic exercise training improves cognition (8), considerably less is known about the impact of free-living or real-world activity on cognitive aging. In middle-aged and older adults, higher self-reported physical activity was associated with an attenuated decrease in processing speed with age (9). Similarly, increased habitual self-reported physical activity was associated with a decreased decline in executive function in older adults with Alzheimer's disease (7). However, the influence of habitual activity, and specifically the intensity of free-living activity, on executive functions in healthy older adults remains understudied.

Strategies to help individuals incorporate more, moderate and vigorous intensity activity as part of their day-to-day life have been promoted. These include concepts such as “exercise snacks” and intermittent lifestyle physical activity (ILPA). Exercise snacks refer to brief (i.e., ∼60 s), planned periodic bouts of physical activity throughout the day to replace a singular longer duration (10). Exercise snacking has been associated with improved physical function and balance in pre-frail older adults (11), muscle function in healthy older adults (12), and cardiovascular fitness in young adults (13). Although practically similar, ILPA reflects brief and *unplanned* bouts (<60 s) of physical activity of varying intensity integrated into an individual's daily routine (14). ILPA is a relatively novel concept in physical activity research, but preliminary findings suggest that more vigorous-ILPA may contribute to a reduction in all-cause (15), cancer-specific, and cardiovascular disease mortality (16, 17). We have previously documented that more time spent in moderate physical activity was associated with better cognitive flexibility and greater oxygenation of the prefrontal cortex during a Stroop Task (18). However, the impact of physical activity patterns and ILPA intensity on executive function is unclear. While epidemiological research documents a favorable impact of vigorous-ILPA on mortality (16, 17), it is unclear whether light- or moderate-intensity ILPA may also be linked with health benefits or whether more time spent engaging in ILPA leads to cognitive benefits in older adults.

The main objective of this study was to determine the relationship between ILPA with executive function as assessed via a Trail-Making-Task in cognitively healthy older adults. It was hypothesized that more time spent in moderate-ILPA or -vigorous-ILPA would be associated with faster reaction times on the Trail-Making-Task.

## Methods

### Participants

This cross-sectional study recruited 40 older adults, >55 years (26 female) from the Acadia University Active Aging program. A sub-sample (*n* = 32) of the activity monitor data have been previously presented (18). However, the ILPA and Trail-Making-Task outcomes were not presented. This study answers a novel, independent research question. In the absence of a well-informed effect size, an estimated moderate effect size (*r* = 0.50), a bivariate correlational model calculated that a minimum of 29 participants were needed assuming a two-tailed, *α* = 0.05 and *β* = 80% power (19). Participants had no physical limitations to exercise and a resting blood pressure <140/90 mmHg and resting heart rate <100 beats/min. All participants were healthy and had normal-to-corrected vision. None of the participants had a history of neurological or psychiatric disorders, color blindness, surgery with general anesthesia during the previous 6 months, involuntary tremors, epilepsy or drug/alcohol problems. Some participants were taking medications for hypothyroidism (Synthroid, *n* = 4) and high blood pressure (Teveten, *n *= 1 and Adalat XL, *n = *1). Participants were excluded if they scored <25 out of 30 on the Mini-Mental State Examination (average: 29.4 ± 1.2). Research Ethics Board approval was obtained from Dalhousie University and Acadia University. Participants were informed of the methods and study design verbally and in writing before providing written informed consent.

### Anthropometrics and Trail-Making-Task measurements

Height and weight were measured using a calibrated stadiometer and physician's scale (Health-O-Meter, McCook Il, USA) to the nearest 0.5-cm and 0.1-kg respectively. Body mass index was calculated as body mass (kg) ÷ height (m)^2^.

The Trail-Making-Task is a widely recognized and validated cognitive assessment tool used in research (20). Participants completed both parts of the test: Part A (Trail A) and Part B (Trail B). Part A assess processing speed and involves participants drawing connecting lines between numbers in ascending order. Participants were instructed to, “Please take the pencil and draw a line from one number to the next, in order. Start at 1 [point to the number], then go to 2 [point], then go to 3 [point], and so on. Please try not to lift the pen as you move from one number to the next. Work as quickly and accurately as you can” (21). Emphasis was placed on both speed and accuracy. Participants were encouraged to correct any errors, and the total time required to complete the task was recorded in seconds. Part B of the Trail-Making-Task (Trail B) evaluated cognitive flexibility and switching ability. Participants were given the same instructions as in Trail A but had to alternate between numbers in ascending order and letters in alphabetical order (1-A-2-B-3-C, etc.). The time required to complete Trail B was also measured in seconds.

Before administering the standardized version of the test, participants were provided with a brief practice trial to familiarize themselves with the task requirements. This practice trial was given prior to each part of the test to ensure that participants understood the instructions and could perform the task accurately.

### Free-living activity monitoring

The activPAL inclinometer (V3, Pal Technologies LTD. Glasgow, UK) was used to objectively measure physical activity and sedentary time. The activPAL is a valid measure of free-living posture (22) and physical activity (23). All participants wore the activPAL 24-h/day for 5–7 days (6.2 ± 1.8 days) based on previous wear time recommendations (24). The activPAL was waterproofed and secured using a nitrile finger cot and a transparent medical dressing to the midline of their right thigh, one third of the way between the hip and knee (25).

The raw activPAL data were exported into PAL analysis (version 5.8.5) for data processing, this program produced a range of activity summaries, including an events and a 15s epoch file. Further processing of these summaries was conducted using a customized MATLAB program (MathWorks, Portola Valley, CA, USA) that produced daily averages of time awake, standing time, and sedentary time. An additional, openly available, LabVIEW (National Instruments, Austin, TX, USA) program determined time spent in each physical activity intensity via step rate thresholds determined based on body mass index (26).

### Intermittent lifestyle physical activity

The activPAL provides an Events XYZ.csv file, which classifies raw acceleration counts into postural activities (i.e., sedentary, standing, or stepping) and includes timestamps of activity, tri-axial acceleration profiles, and the duration of each postural bout. Another customized LabVIEW bout-cadence program was created to calculate time spent in various intensities of physical activity. Sixty-second bouts were chosen based on the definition provided in a previous ILPA study (14). Using body mass index-tailored step rate thresholds (27), the program sorted through the Events XYZ file to categorize stepping bouts as light physical activity, moderate physical activity, or vigorous physical activity from the average cadence recorded via the activPAL and the bout duration. Light physical activity was characterized as anything < 108.2 ± 2.4 steps/min, moderate physical activity as anything between 108.2 ± 2.4 and 134.5 ± 4.6 steps/min, and vigorous physical activity was anything >134.5 ± 4.6 steps/min. The program exported a.csv summary file which included daily totals of the frequency and duration (in minutes) in light physical activity, moderate physical activity, and, vigorous physical activity for <60-s bouts, ≥60-s bouts, and all bouts. ILPA is classified as short bouts <60 s, bouts ≥60 s used for exclusively longer physical activity, total physical activity includes both short and long bouts.

### Statistical analysis

All data were assessed for normality using a Shapiro-Wilk test and data were analyzed using non-parametric statistical tests. Specifically, the relationship between each physical activity intensity vs. Trail A and Trail B completion times were analyzed using Spearman's Rank correlations. Interactions between each ILPA intensity with age or sex were determined for both Trail A and Trail B times. Exploratory analyses indicated no significant interactions were observed (age × ILPA: all, *p *> 0.15; sex × ILPA: all *p > *0.06), indicating that correlational analyses can be conducted on the pooled sample and that neither sex nor age moderated this relationship in this specific study. All statistics were completed in SPSS Version 28.0 (IBM, NY). Statistical significance was accepted as *p *< 0.05. All data are presented as means ± standard deviations.

## Results

Data from 40 older adults (26 females) with an average age of 68 ± 6 years (56–83), a body mass index of 26.6 ± 4.3 kg/m^2^, average heart rate of 69 ± 10 beats/min, an average systolic blood pressure of 124 ± 11 mmHg and diastolic blood pressure of 71 ± 9 mmHg were included in the current study (Table 1). Participants accumulated 9.1 ± 1.6 h/day of sedentary time and 6.3 ± 1.4 h/day of standing time. The completion times for Trail A and Trail B were 25.8 ± 8.2 s and 63.2 ± 26.2 s, respectively. Total-moderate physical activity, moderate-ILPA, and exclusively longer bouts (≥60 s) of moderate physical activity were engaged in for an average of 20.1 ± 16.0 min/day, 3.2 ± 3.2 min/day and 16.9 ± 15.2 min/day, respectively. Light physical activity, on average, was engaged in for 6.5% of the day, moderate physical activity for 1.4% of the day and vigorous physical activity for 0.02% of the day.

**Table 1:** Participant descriptive characteristics, habitual posture, and physical activity outcomes.

Variable	Participant (*n *= 40)
Age (years)	68 ± 6 (56–83)
Sex (males, females)	14, 26
Height (cm)	165.3 ± 9.8 (146.0–183.0)
Weight (kg)	71.2 ± 13.7 (41.0–107.0)
Body mass index (kg/m^2^)	26.6 ± 4.3 (19.4–40.6)
MMSE (score/30)	29.4 ± 1.2 (26–30)
Step count (steps/day)	9,203 ± 3,439 (3,772–19,694)
Sedentary time (hours/day)	9.1 ± 1.6 (2.6–10.7)
Standing time (hours/day)	6.3 ± 1.4 (3.5–11.5)
Light physical activity-total (min/day)	93.0 ± 29.5 (35.3–155.4)
Total-moderate physical activity (min/day)	20.1 ± 16.1 (0.2–70.9)
Vigorous physical activity-total (min/day)	2.4 ± 3.1 (0.1–12.9)
Light physical activity duration ≥60 s (min/day)	22.1 ± 14.5 (4.1–67.5)
Moderate physical activity duration ≥60 s (min/day)	16.9 ± 15.2 (0.0–69.0)
Vigorous physical activity duration ≥60 s (min/day)	2.1 ± 2.9 (0.1–12.7)
Light-ILPA duration (min/day)	70.9 ± 22.8 (31.2–120.7)
Moderate-ILPA duration (min/day)	3.2 ± 3.2 (0.05–13.6)
Vigorous-ILPA duration (min/day)	0.3 ± 0.7 (0.03–2.9)
Light-ILPA frequency (bouts/day)	363.0 ± 115.3 (8.9–617.6)
Moderate-ILPA frequency (bouts/day)	24.0 ± 19.1 (1.4–83.3)
Vigorous-ILPA frequency (bouts/day)	17.3 ± 6.9 (1.7–42.1)

Data presented as means ± SD (range).

SBP, systolic blood pressure; DBP, diastolic blood pressure; MMSE, mini-mental state exam.

**Figure 1:**
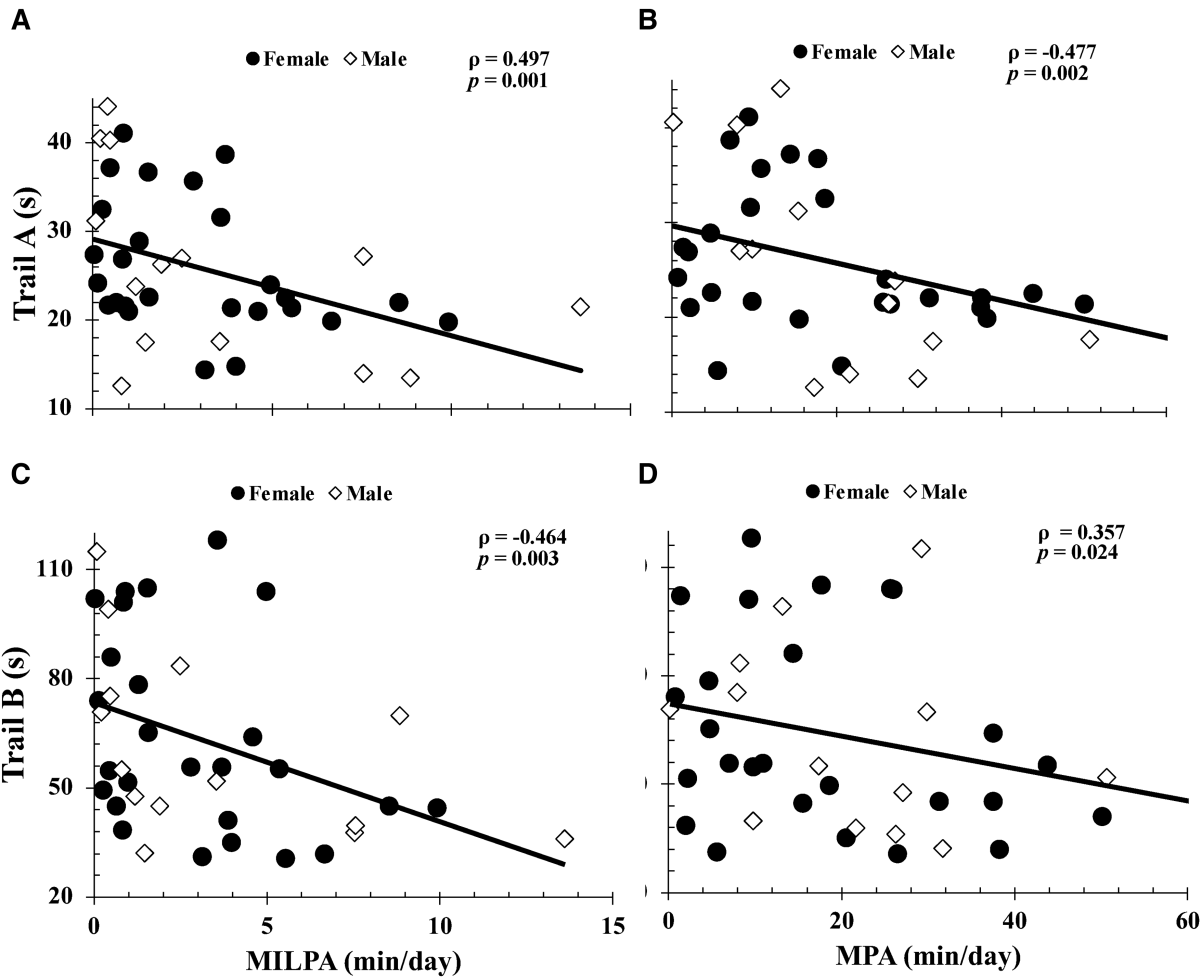
Relationships between trail A completion time with moderate intermittent lifestyle physical activity (moderate-ILPA) duration (**A**), moderate physical activity duration (**B**), and between trail B completion time vs. moderate-ILPA duration (**C**) and moderate physical activity duration (**D**). Relationships were determined via Spearman's Rank Correlations. Data are presented for *n *= 40.

**Figure 2:**
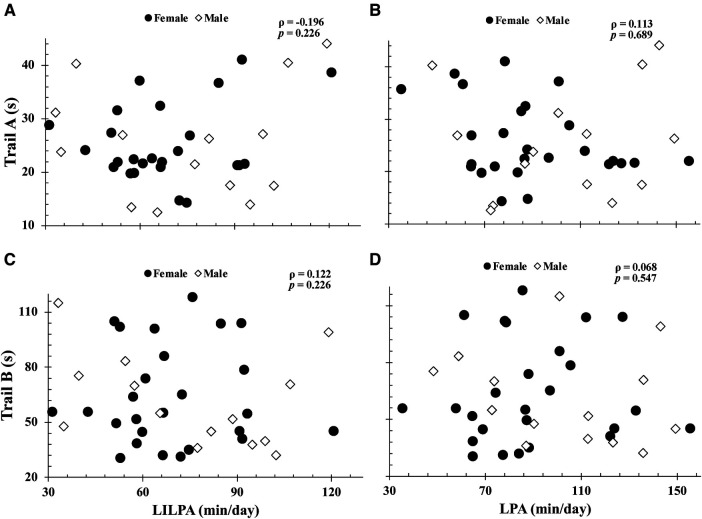
Relationships between trail A completion time with light intermittent lifestyle physical activity (light-ILPA) duration (**A**), light physical activity duration (**B**), and between trail B completion time vs. light-ILPA duration (**C**) and light physical activity duration (**D**). Relationships were determined via Spearman's Rank Correlations. Data are presented for *n *= 40.

**Figure 3:**
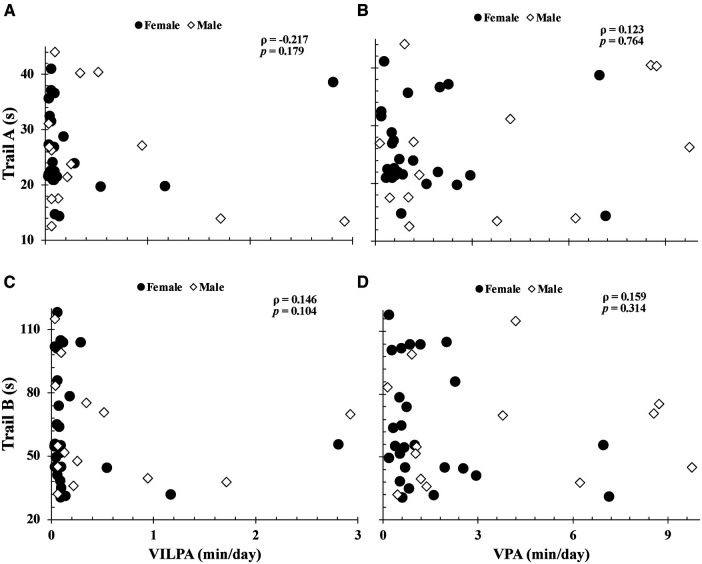
Relationships between trail A completion time with vigorous intermittent lifestyle physical activity (vigorous-ILPA) duration (**A**), vigorous physical activity duration (**B**), and between trail B completion time vs. vigorous-ILPA duration (**C**) and vigorous physical activity duration (**D**). Relationships were determined via Spearman's Rank Correlations. Data are presented for *n *= 40.

More time spent engaged in total-moderate physical activity and moderate-ILPA were associated with faster Trail A (total-moderate physical activity: *ρ* = −0.48; moderate-ILPA: *ρ* = −0.50; both, *p *< 0.003) and Trail B completion times (total-moderate physical activity: *ρ* = −0.36; moderate-ILPA: *ρ* = −0.46; both, *p *< 0.020) (Figure 1). In contrast, time spent in moderate physical activity bouts lasting ≥60 s (16.9 ± 15.2 min/day) was negatively correlated with Trail A completion time (*ρ = *−0.38 *p = *0.02), but not with Trail B (*ρ *= −0.21 *p *= 0.20). As seen in Figures 2, 3, neither total- or ILPA for vigorous or light physical activity, were associated with Trail A or Trail B performance (all, *p *> 0.18).

## Discussion

The purpose of this study was to examine the relationship between intensity based ILPA durations and executive function in cognitively healthy older adults. Consistent with our hypothesis, more time spent in total-moderate physical activity and moderate-ILPA was associated with faster completion times on both Trail conditions. However, unexpectedly, vigorous-ILPA was not associated with Trail task performance (Figure 3). Studying the associations between physical activity intensity and executive function is of importance with advancing age, as individuals become more susceptible to cognitive decline. The findings of this study provide support for the beneficial impact of brief periods of moderate physical activity on executive function in older adults that may enhance cognitive well-being and positions easy-to-do activity as an important part of healthy cognitive aging.

A decline in executive function is an early predictor for a range of neurological conditions (3). Our study demonstrated that both moderate-ILPA and total-moderate physical activity were associated with higher processing speed (Trail A) and cognitive flexibility (Trail B). In contrast longer bouts (≥60 s) of moderate physical activity were not associated with Trail B performance. These findings demonstrate that the association of faster reaction times and thus, a more favorable executive function with physical activity is primarily associated with ILPA (<60s bouts), not exclusively longer bouts (>60 s). The relationship with both Trail A and Trail B indicates that moderate physical activity may be beneficial for both lower-level (e.g., processing speed) and higher-order cognitive processes (e.g., cognitive flexibility). It is important to note that while the results indicate that moderate ILPA may be beneficial, total moderate physical activity showed the same benefits. Therefore, this study is adding to the previous literature as it is well established that moderate physical activity can improve cognition, however, short bouts (ILPA) have been understudied. These observations are encouraging as brief periods of moderate physical activity may be more feasible to conduct than longer bouts. Furthermore, engaging in higher intensity physical activity is often more difficult for some older adults (28). Walking is the most common form of physical activity among older adults (29) and brisk walking is typically conducted at a moderate intensity (∼100–110 steps/min) (30). Therefore, the present observations may inform the design of future movement interventions for older adults that target improvements in executive function.

Increased physical activity is associated with improved cognitive function and decreased cognitive decline with age for older adults with, and without impairment (31). Research has found an association between objectively measured higher intensity physical activity and better cognitive function, however, there was no association found between increased total physical activity duration and increased cognitive function (32). Additionally, increased self-reported physical activity intensity was associated with reduced cognitive decline in older men (33). Accordingly, it may not be longer total duration of physical activity, but rather, higher intensity that is important. The prefrontal cortex is recognized as the key structure for executive function (34) and it has been demonstrated that physical activity increases both blood oxygenation (35) and arousal (36) in the prefrontal cortex. Furthermore, it has been demonstrated that engaging in more moderate physical activity is linked to preserving volume of the dorsolateral prefrontal cortex with age, compared to those who are insufficiently active (37). Accordingly, the relationships observed may be due to the positive impact of moderate-ILPA on brain structures associated with executive function. Other research indicates that more physical activity increases levels of brain-derived neurotrophic factor, which is associated with improved brain function and plasticity (36). In addition to these prefrontal cortex processes and improved neuroplasticity, exercise has demonstrated the potential to reduce neuroinflammation, which plays a role in the development of neurological disorders with declining executive function, such as Alzheimer's (38). All factors mentioned could explain the mechanistic links between ILPA and executive function, and the protective nature of exercise on cognitive health. Future work incorporating these mechanistic measures are warranted.

As reported with the World Health Organization guidelines, moderate-to-vigorous physical activity is recommended for improved health in older adults (6). Our results are consistent with this, as our average for total moderate physical activity was approximately 150 min/week, and more total moderate physical activity was associated with faster completion times for both Trail A and B tasks. While moderate-ILPA only represented 1.4% of participants daily time, it was also associated with faster completion times on both tasks, as such, future interventional research on increasing moderate-ILPA is warranted. While the specific amount of moderate-ILPA that translates to cognitive benefits are unclear, more moderate-ILPA in general is associated with executive function. Vigorous physical activity was not associated with either of the Trail-Making-Tasks. It should be noted that in our participants, the duration of vigorous physical activity was much lower than that of moderate and light physical activity, thus, the minimal range of time spent in vigorous physical activity may be insufficient to reveal a correlational relationship. Additionally, the moderate-ILPA data has a much larger variance, which is beneficial for correlational analysis, compared to the vigorous-ILPA data (see Figures 1, 3). Interventional models aimed at promoting better executive functions among cognitively healthy older adults should consider moderate physical activity, and specifically studying the impact of integrating more brief periods of moderate physical activity into their lifestyle on cognitive outcomes.

The primary limitation of the study is its cross-sectional design and therefore cannot determine causality. However, the work is important in directing future intervention studies and applies novel objectively measured activity outcomes. Our participants were cognitively healthy older adults, and our observations may not be extrapolated to adults with cognitive impairments who may be less active (39). In these populations, there is a possibility that more light physical activity or less sedentary time may be useful for individuals with cognitive impairments. The definition for older adults being >55 or >65 years is ambiguous, additionally, these results may not be generalized to older adults above the age of 85. This study did not investigate the mechanistic links of the pathways that might explain the association between ILPA and executive function, or the specific type of physical activity conducted by participants. However, our study is strengthened by its consideration of ILPA, a novel approach to physical activity, and its relationship with executive functions in older adults. Our observations provide valuable insights to a relatively understudied perspective regarding the most optimal physical activity intensity and pattern for healthy cognition.

Among cognitively healthy older adults, engaging in more moderate-ILPA and more total-moderate physical activity was associated with faster completion times in the Trail-Making-Task, indicating better executive function. Given that impairments in executive functions are the initial characteristics of cognitive decline, strategies that investigate the impact of- and promote more brief periods of moderate physical activity, may be easy to integrate as lifestyle behaviors that improve cognitive health.

## Data Availability

The raw data supporting the conclusions of this article will be made available by the authors, upon reasonable request.

## References

[1] Bishop NA Lu T Yankner BA. Neural mechanisms of ageing and cognitive decline. Nature. (2010) 464:529–35. 10.1038/nature08983 20336135 PMC2927852

[2] Gilbert SJ Burgess PW. Executive function. Curr Biol. (2008) 18:110–4. 10.1016/j.cub.2007.12.014 18269902

[3] Levy G Jacobs DM Tang MX Côté LJ Louis ED Alfaro B Memory and executive function impairment predict dementia in Parkinson’s disease. Mov Disord. (2002) 17:1221–6. 10.1002/mds.10280 12465060

[4] Fine EM Delis DC Wetter SR Jacobson MW Jak AJ Mcdonald CR Cognitive discrepancies versus APOE genotype as predictors of cognitive decline in normal-functioning elderly individuals: a longitudinal study. Am J Geriatr Psychiatry. (2008) 16(5):366–74. 10.1097/JGP.0b013e3181629957 18448849 PMC3050584

[5] Tseng CN Gau BS Lou MF. The effectiveness of exercise on improving cognitive function in older people: a systematic review. J Nurs Res. (2011) 19:119–31. 10.1097/JNR.0b013e3182198837 21586989

[6] Bull FC Al-Ansari SS Biddle S Borodulin K Buman MP Cardon G World Health Organization 2020 guidelines on physical activity and sedentary behaviour. Br J Sports Med. (2020) 54:1451–62. 10.1136/bjsports-2020-102955 33239350 PMC7719906

[7] Farina N Tabet N Rusted J. The relationship between habitual physical activity status and executive function in individuals with Alzheimer’s disease: a longitudinal, cross-lagged panel analysis. Neuropsychol Dev Cogn B Aging Neuropsychol Cogn. (2016) 23:234–52. 10.1080/13825585.2015.1080213 26330266

[8] Fernandes RM Correa MG dos Santos MAR Almeida APCPSC Fagundes NCF Maia LC The effects of moderate physical exercise on adult cognition: a systematic review. Front Physiol. (2018) 9:667. 10.3389/fphys.2018.00667 29937732 PMC6002532

[9] Angevaren M Vanhees L Nooyens ACJ Wendel-Vos CGW Verschuren WMM. Physical activity and 5-year cognitive decline in the doetinchem cohort study. Ann Epidemiol. (2010) 20:473–9. 10.1016/j.annepidem.2010.03.007 20470975

[10] Francois ME Baldi JC Manning PJ Lucas SJE Hawley JA Williams MJA “Exercise snacks” before meals: a novel strategy to improve glycaemic control in individuals with insulin resistance. Diabetologia. (2014) 57:1437–45. 10.1007/s00125-014-3244-6 24817675

[11] Western MJ Welsh T Keen K Bishop V Perkin OJ. Exercise snacking to improve physical function in pre-frail older adult memory clinic patients: a 28-day pilot study. BMC Geriatr. (2023) 23:471. 10.1186/s12877-023-04169-6 37542234 PMC10403822

[12] Perkin OJ McGuigan PM Stokes KA. Exercise snacking to improve muscle function in healthy older adults: a pilot study. J Aging Res. (2019) 2019:7516939. 10.1155/2019/7516939 31687210 PMC6794984

[13] Jenkins EM Nairn LN Skelly LE Little JP Gibala MJ. Do stair climbing exercise “snacks” improve cardiorespiratory fitness? Appl Physiol Nutr Metab. (2019) 44:681–4. 10.1139/apnm-2018-0675 30649897

[14] Stamatakis E Huang BH Maher C Thøgersen-Ntoumani C Stathi A Dempsey PC Untapping the health enhancing potential of vigorous intermittent lifestyle physical activity (VILPA): rationale, scoping review, and a 4-pillar research framework. Sports Med. (2021) 51:1–10. 10.1007/s40279-020-01368-8 33108651 PMC7806564

[15] Ahmadi MN Hamer M Gill JMR Murphy M Sanders JP Doherty A Brief bouts of device-measured intermittent lifestyle physical activity and its association with major adverse cardiovascular events and mortality in people who do not exercise: a prospective cohort study. Lancet Public Health. (2023) 8:e800–10. 10.1016/S2468-2667(23)00183-4 37777289

[16] Stamatakis E Ahmadi MN Gill JMR Thøgersen-Ntoumani C Gibala MJ Doherty A Association of wearable device-measured vigorous intermittent lifestyle physical activity with mortality. Nat Med. (2022) 28:2521–9. 10.1038/s41591-022-02100-x 36482104 PMC9800274

[17] Stamatakis E Ahmadi MN Friedenreich CM Blodgett JM Koster A Holtermann A Vigorous intermittent lifestyle physical activity and cancer incidence among nonexercising adults: the UK biobank accelerometry study. JAMA Oncol. (2023) 9:1255–9. 10.1001/jamaoncol.2023.1830 37498576 PMC10375384

[18] O’Brien MW Kimmerly DS Mekari S. Greater habitual moderate-to-vigorous physical activity is associated with better executive function and higher prefrontal oxygenation in older adults. Geroscience. (2021) 43:2707–18. 10.1007/s11357-021-00391-5 34081258 PMC8602604

[19] Faul F Erdfelder E Buchner A Lang A-G. Statistical power analyses using G*Power 3.1: tests for correlation and regression analyses. Behav Res Methods. (2009) 41:1149–60. 10.3758/BRM.41.4.1149 19897823

[20] Sánchez-Cubillo I Periáñez JA Adrover-Roig D Rodríguez-Sánchez JM Ríos-Lago M Tirapu J Construct validity of the trail making test: role of task-switching, working memory, inhibition/interference control, and visuomotor abilities. J Int Neuropsychol Soc. (2009) 15:438–50. 10.1017/S1355617709090626 19402930

[21] Tombaugh T. Trail making test A and B: normative data stratified by age and education. Arch Clin Neuropsychol. (2004) 19:203–14. 10.1016/S0887-6177(03)00039-8 15010086

[22] O’Brien MW Wu Y Petterson JL Bray NW Kimmerly DS. Validity of the ActivPAL monitor to distinguish postures: a systematic review. Gait Posture. (2022) 94:107–13. 10.1016/j.gaitpost.2022.03.002 35276456

[23] Wu Y Petterson JL Bray NW Kimmerly DS O’Brien MW. Validity of the activPAL monitor to measure stepping activity and activity intensity: a systematic review. Gait Posture. (2022) 97:165–73. 10.1016/j.gaitpost.2022.08.002 35964334

[24] Hart TL Swartz AM Cashin SE Strath SJ. How many days of monitoring predict physical activity and sedentary behaviour in older adults? Int J Behav Nutr Phys Act. (2011) 8:62. 10.1186/1479-5868-8-62 21679426 PMC3130631

[25] Edwardson CL Winkler EAH Bodicoat DH Yates T Davies MJ Dunstan DW Considerations when using the activPAL monitor in field-based research with adult populations. J Sport Health Sci. (2017) 6:162–78. 10.1016/j.jshs.2016.02.002 30356601 PMC6188993

[26] Johns JA Frayne RJ Goreham JA Kimmerly DS O’Brien MW. The bout cadence method improves the quantification of stepping cadence in free-living conditions. Gait Posture. (2020) 79:96–101. 10.1016/j.gaitpost.2020.04.014 32387810

[27] O’Brien MW Kivell MJ Wojcik WR D’Entremont GR Kimmerly DS Fowles JR. Influence of anthropometrics on step-rate thresholds for moderate and vigorous physical activity in older adults: scientific modeling study. JMIR Aging. (2018) 1:e12363. 10.2196/12363 31518246 PMC6715008

[28] Chastin SFM Buck C Freiberger E Murphy M Brug J Cardon G Systematic literature review of determinants of sedentary behaviour in older adults: a DEDIPAC study. Int J Behav Nutr Phys Act. (2015) 12:127. 10.1186/s12966-015-0292-3 26437960 PMC4595239

[29] Mobily KE. Walking among older adults. World Leis J. (2014) 56:130–40. 10.1080/16078055.2014.903725

[30] Grant PM Dall PM Mitchell SL Granat MH. Activity-monitor accuracy in measuring step number and cadence in community-dwelling older adults. J Aging Phys Act. (2008) 16(2):201–14. 10.1123/japa.16.2.201 18483442

[31] Busse AL Gil G Santarém JM Filho WJ. Physical activity and cognition in the elderly: a review. Dementia e Neuropsychologia. (2009) 3:204–8. 10.1590/S1980-57642009DN30300005 PMC5618974 29213629

[32] Brown BM Peiffer JJ Sohrabi HR Mondal A Gupta VB Rainey-Smith SR Intense physical activity is associated with cognitive performance in the elderly. Transl Psychiatry. (2012) 2:e191. 10.1038/tp.2012.118 23168991 PMC3565765

[33] Van Gelder BM Tijhuis MAR Kalmijn S Giampaoli S Nissinen A Kromhout D. Physical activity in relation to cognitive decline in elderly men: the FINE Study. Neurology. (2004) 63(12):2316–21. 10.1212/01.wnl.0000147474.29994.35 15623693

[34] Funahashi S Andreau JM. (2013). Prefrontal cortex and neural mechanisms of executive function. Available online at: https://repository.kulib.kyoto-u.ac.jp (Accessed December 1, 2023). 10.1016/j.jphysparis.2013.05.001 23684970

[35] Mekari S Dupuy O Martins R Evans K Kimmerly DS Fraser S The effects of cardiorespiratory fitness on executive function and prefrontal oxygenation in older adults. Geroscience. (2019) 41:681–90. 10.1007/s11357-019-00128-5 31728899 PMC6885073

[36] Cotman CW Berchtold NC. (2002). Exercise: a behavioural intervention to enhance brain health and plasticity. Available online at: http://tins.trends.com 10.1016/s0166-2236(02)02143-4 12086747

[37] Northey JM Rattray B Pumpa KL Pryor DJ Fraser MA Shaw ME Objectively measured physical activity is associated with dorsolateral prefrontal cortex volume in older adults. Neuroimage. (2020) 221:117150. 10.1016/j.neuroimage.2020.117150 32668298

[38] Mee-Inta O Zhao ZW Kuo YM. Physical exercise inhibits inflammation and microglial activation. Cells. (2019) 8:691. 10.3390/cells8070691 31324021 PMC6678635

[39] Taylor JS Demers SM Vig EK Borson S. The disappearing subject: exclusion of people with cognitive impairment and dementia from geriatrics research. J Am Geriatr Soc. (2012) 60:413–9. 10.1111/j.1532-5415.2011.03847.x 22288835

